# Art healing or art therapy? Untangling conceptual confusion in China and advancing a pathway toward community institutionalization

**DOI:** 10.3389/fpsyg.2025.1708156

**Published:** 2026-01-12

**Authors:** Zichen Ke, Xiang Meng, Muhizam Mustafa

**Affiliations:** 1 School of the Arts, Universiti Sains Malaysia, Penang, Malaysia; 2 School of the Art and Design, Anyang Institute of Technology, Anyang, Henan, China

**Keywords:** art healing, art therapy, conceptual confusion, institutionalization, professionalization

## Abstract

The field of art therapy in China is currently facing severe conceptual confusion between art therapy and art healing. While international scholarship has widely noted the inherently fluid boundaries among these practices, this paper argues that in China, where an institutional vacuum exists, such fluidity has resulted not in productive debate but in a conceptual conflation. This paper examines how such confusion manifests across academic research, educational practices, and the commercial market, arguing that it has led to de-professionalization and hindered the sustainable development of community-based mental health services. Drawing on internationally recognized practice elements of community art therapy, this paper uses a conceptual analysis approach to critically synthesize literature, policy documents, and international professional standards, and proposes that China should establish a threefold institutional pathway comprising certification and regulatory mechanisms, interdisciplinary education, and policy integration. This framework would help delineate conceptual boundaries, advance the professionalization of art therapy, and promote its institutionalization within community settings, thereby enabling art therapy to contribute effectively to public mental health services and social integration.

## 1 Introduction

Since its emergence in the early twentieth century in the West, art therapy has gradually been incorporated into the fields of public health and social wellbeing, and has evolved into non-clinical forms of practice such as community art therapy (Goldstein Nolan and Mumpton, 2024). This practice uses artistic creation as a medium and relies on non-verbal forms of expression to promote participants' mental health, helping to relieve emotional stress, distract from pain, enhance self-awareness, and rebuild trust and communication with others (Malchiodi, 2011; O'Neill and Moss, 2015). Its target populations include a wide range of groups such as children, older adults, military personnel, survivors of violence and trauma, and individuals with dementia or Alzheimer's disease (American Art Therapy Association, n.d.).

In Western contexts, community art therapy must be delivered by art therapists who hold relevant degrees or professional licenses, and it is commonly practiced in schools, care homes, community health centers, and non-profit organizations, often supported by public funding or health insurance (American Art Therapy Association, n.d.; Hinz, 2019). Compared with art therapy conducted in clinical settings, community art therapy places greater emphasis on accessibility, public orientation, and social integration, providing psychological support and emotional nourishment for both individuals and communities (Stuckey and Nobel, 2010). Nevertheless, in practice, it is still common to find such professional therapeutic activities being broadly or interchangeably referred to as art healing or arts in health, which blurs their boundaries with art therapy (Sonke et al., 2009; Zarobe and Bungay, 2017). In the literature on arts in health, the term art healing is often used as an umbrella for non-clinical arts-based activities that seek to enhance wellbeing (Davies and Clift, 2022; Fancourt and Finn, 2019), and is therefore not equivalent to art therapy in the professional sense. These activities include workshops and programmes led by artists or community practitioners rather than registered therapists (Sonke et al., 2009; Zarobe and Bungay, 2017; Huss, 2013), as well as a wide range of informal healing activities commonly found in community and digital spaces. Such practices can play a valuable role in emotional support and health promotion. However, they do not constitute art therapy or psychotherapy unless they are embedded in a treatment framework and led by trained art therapists (Davies and Clift, 2022; Sonke et al., 2009; Zarobe and Bungay, 2017). The key distinction lies not in where these practices take place, but in whether they are grounded in psychotherapeutic theory, assessment and ethical obligations, and whether they are delivered by practitioners trained and licensed as art therapists (Malchiodi, 2011; Huss and Bos, 2018).

The challenge of further distinguishing art therapy from broader arts-based practices arises from both their experiential overlaps and their inherent theoretical fluidity. On the one hand, non-clinical arts activities can mobilize processes such as emotional regulation, symbolic expression, stress reduction, and collective meaning-making, and such effects have been documented in community art projects, healthcare settings, workplace wellbeing programs, and post-disaster or crisis interventions (Huss, 2013; Huss and Sarid, 2014; Huss et al., 2015; Bojner Horwitz et al., 2017; Huss et al., 2016; Huss and Havsteen-Franklin, 2023). These effects closely resemble therapeutic processes and therefore lead participants and practitioners alike to describe such activities as “healing.” On the other hand, scholarship increasingly frames community art, arts in health and community art therapy as a fluid continuum shaped by shared social and relational dynamics, where practices frequently pursue therapeutic, educational and social-change aims simultaneously (Shefi et al., 2022; Kapitan, 2010). This inherent conceptual fluidity means that even in Western contexts with established professional frameworks, the boundary remains debated. Crucially, however, the implications of this fluidity depend on the presence of stabilizing institutional structures. In their absence, as in China, these already-contested concepts do not sustain productive debate but undergo a conceptual conflation, merging into a single, ambiguous and commercially driven category of “healing,” which further exacerbates confusion and hinders professional development.

The American Art Therapy Association (AATA), as one of the most authoritative professional bodies for art therapy worldwide, explicitly states that only licensed mental health professionals who have received systematic training in psychological theories and art therapy, obtained a master's degree or higher, and hold credentials as Registered Art Therapists (ATR/ATR-BC) are qualified to practice art therapy (American Art Therapy Association, n.d.). Similar professional certification systems have also been widely established in countries such as Canada, the United Kingdom, Australia, and New Zealand. Practitioners are required to complete prescribed training, clinical internships, and supervised practice, and to be certified by national or professional regulatory bodies before being legally eligible to practice, such as the Canadian Art Therapy Association (CATA), the British Association of Art Therapists (BAAT), and the Australian, New Zealand and Asian Creative Arts Therapies Association (ANZACATA) (British Association of Art Therapists, n.d.; Canadian Art Therapy Association, n.d.; New Zealand and Asian Creative Arts Therapies Association, n.d.) (see Table 1).

**Table 1:** Professional certification systems for art therapists in western countries.

**Country**	**Professional credential**	**Academic requirement**	**Certifying body**	**Key notes**
United States	Registered Art Therapist (ATR)/Board Certified Art Therapist (ATR-BC)	Master's degree or above in art therapy	Art Therapy Credentials Board (ATCB)	Completion of required art & psychology prerequisites
United Kingdom	State-Registered Art Psychotherapist (HCPC Registration)	Master's degree in art psychotherapy	Health and Care Professions Council (HCPC); British Association of Art Therapists (BAAT)	Two-year programmes with clinical placements
Canada	Registered Canadian Art Therapist (RCAT)	Master's degree in a related field plus professional training	Canadian Art Therapy Association (CATA)	Completion of clinical internship & supervised practice
Australia & New Zealand	Registered Art Therapist	ANZACATA-recognized postgraduate programme	Australian, New Zealand & Asian Creative Arts Therapies Association (ANZACATA)	Completion of an accredited Master's program is a key route to becoming a professional member

Although terminological overlap also appears in Western practice, it operates within a well-regulated system. It therefore represents a conceptual generalization rather than a regulatory ambiguity (Sonke et al., 2009). It is important to note that China currently lacks a regulatory framework, professional certification mechanisms, and unified educational standards for art therapy. In contrast, this institutional vacuum has meant that the direct adoption of these terms has further intensified the misalignment between concepts and practice (Guo, 2024). Especially after the COVID-19 pandemic, China has seen a rapid rise of non-clinical, psychologically soothing art practices promoted on social media under labels such as art healing, mind-body-spirit healing, and experiential art. These activities are predominantly market-oriented and profit-driven, and are commonly offered through commercial workshops, training courses and grassroots organizations (Cui and Wang, 2023). Although these practices partly respond to the growing demand for mental health support among the Chinese public living under high-paced and stressful conditions (Feng et al., 2025), they show a strong tendency toward commercialization, individualization, and consumerism. They focus on providing clients with emotional comfort and artistic experience rather than systematic psychological intervention. In the absence of regulatory oversight and industry standards, this orientation has blurred the boundary between art as healing and art as professional therapy, and has deepened terminological confusion in both public perception and academic discourse.

Overall, the conceptual confusion that has emerged during the introduction of art therapy in China has blurred the boundary between art therapy and emotion-oriented art activities, and has hindered the establishment of a systematic framework in the local context. Because there is still no officially recognized professional pathway or standardized system, public understanding of its actual functions, effectiveness, and professional attributes remains highly uncertain, which has further increased the risk of conceptual confusion. Clarifying the concept of art therapy is therefore a crucial prerequisite for developing a systematic pathway for its advancement in China and for promoting its implementation in community practice. Rather than collecting empirical data, this paper uses a conceptual analysis approach to critically synthesize literature, policy documents, and international professional standards in developing a structured framework of discussion. Its aim is to provide theoretical reference and policy insights for advancing the institutional development of community art therapy in China.

The conceptual analysis in this study follows four steps: (1) defining the ideal meanings and distinctions of the core concepts (“art therapy” and “art healing”) within the international context; (2) mapping their actual uses in Chinese academic, educational, and market practices and identifying deviations from the ideal models; (3) analyzing the structural and institutional roots of such deviations; and (4) developing a conceptual pathway to clarify the concepts and reconstruct the institutional system, by drawing on internationally recognized practice elements of community art therapy, and adapting them to the Chinese context.

## 2 Conceptual confusion and institutional absence: the structural challenges of art therapy in China

Although art therapy has gained increasing public attention in China in recent years, its development still faces many structural challenges. According to (Cui and Wang 2023); (Zhang and Ye 2025), art therapy was first introduced to China in the 1990's, while scholarly research on this topic only began to appear in 2015, and (Zhang and Ye 2025) note that the field is still in its initial enlightenment stage. Therefore, conceptual confusion is not merely an isolated academic issue; its influence has permeated and become visible across multiple levels.

### 2.1 Multiple manifestations of conceptual confusion: from academia and education to market practice

In China, the introduction of concepts such as community art, arts in health and community art therapy has taken place without the regulatory, educational and ethical infrastructures that ordinarily give these terms stability and boundaries. As a result, distinctions that remain debated yet institutionally anchored in Western contexts have, in practice, become conceptually conflated into a single loosely framed category. As noted by (Cui and Wang 2023), domestic research has widely adopted “art healing” as a core thematic term. This choice of terminology reflects an initial conceptual deviation, and its ambiguity has systematically undermined the perceived professional legitimacy of the field. While art therapy is rooted in a formalized psychotherapeutic system (Malchiodi, 2011), art healing has, due to its one-sided emphasis on “healing” (Stuckey and Nobel, 2010; Zarobe and Bungay, 2017), been stripped of the therapeutic core within an institutional vacuum, accelerating its drift toward generalization and de-professionalization within the consumer market.

A large number of academic studies, such as (Hou and Zheng 2023), (Zheng 2024), (Ma 2024), (Tang 2025) and (Feng et al. 2025) have conflated art healing with art therapy and have used this assumption as a theoretical foundation for their arguments. Some even explicitly state that art healing and art therapy can sometimes be used interchangeably in research. Such studies generally do not include the core components of psychotherapy, including assessment criteria, intervention procedures and outcome evaluation (Huss, 2013). Their research essence remains primarily grounded in perspectives of art education or cultural creation, and is mostly situated within the disciplines of art and design (Cui and Wang, 2023). This approach has generalized art therapy into a broad emotion-soothing method. The confusion in terminology and the deviation in research orientation have undermined its legitimacy and credibility as a professional form of adjunctive intervention, and have inadvertently amplified conceptual confusion among the public during the process of data collection.

Conceptual confusion is also evident in the field of education. Although some universities have already established relevant degree programs or courses (Guo, 2024), their fragmented development lacks unified professional standards and faculty requirements, which has led to substantial variation in the quality and orientation of such courses. For example, in the core course “Models and Pathways of Art Therapy” described by (Shi 2025), the teaching practice essentially dismantles the professional core of art therapy. The logic of artistic production replaces the participant-centered therapeutic process, and the course tends to emphasize project outcomes and the interpretation of design concepts. However, it lacks essential elements such as relational tracking, psychological support for participants, and ethical safety, which are fundamental competencies required of art therapists (Franklin, 2010). In addition, course information shows that its teaching staff are primarily from art and design backgrounds, with psychology staff only engaged as external advisors. This structure further weakens the psychological foundation of the course and reduces teaching practice to merely maintaining the outward appearance of art therapy, treating art as the central focus while failing to provide students with training that connects to clinical or rehabilitative applications (Donald, 2025; Li, 2024). This suggests that academic institutions themselves have not been able to avoid the pitfalls of conceptual generalization, and that their current models of talent development may be systematically reinforcing local confusion about the professional nature of art therapy.

It should be noted that although some bibliometric studies have suggested that art therapy research in the field of education in China has grown rapidly (e.g., Xing et al., 2024), such studies mainly rely on external information analysis methods such as keyword co-occurrence and lack substantive content-based screening of the literature (Donthu et al., 2021; Klarin, 2024). As a result, they do not clarify whether the included publications involve core elements such as psychological assessment, intervention procedures and ethical standards, nor do they distinguish whether their research subjects are art therapy in the professional sense or art activities oriented toward aesthetic experience. The growth they reveal therefore only reflects an increase in the frequency of term usage rather than substantive progress in the professionalization and scientification of the field.

In addition, at the practical level, conceptual confusion has spread into the consumer market. On social media platforms such as Xiaohongshu (Little Red Book) and Douyin (TikTok China), a large number of commercial art workshops marketed under the label of “healing” have packaged art activities as paid services for emotional relief. The qualifications of practitioners are often unclear, and there is no industry entry threshold, which has further fuelled public misunderstanding of the professional nature of art therapy. As a result, it is often perceived simply as a short-term, consumer-oriented emotional experience rather than as a serious form of psychological intervention. This conceptual generalization, which extends from academia to the market, essentially reflects the lack of institutional support in China for the introduction of art therapy, and this absence constitutes the root cause of its deeper structural difficulties.

### 2.2 Institutional deficiency: the root cause of conceptual confusion

These chaotic phenomena are not accidental. Their root cause lies in the fact that, when introducing Western concepts, China has bypassed the crucial process of institutional internalization, resulting in systemic deficiencies across the dimensions of certification, education, policy, and culture.

Firstly, the absence of a certification and regulatory system is the most fundamental institutional deficiency (Guo, 2024). China lacks any national-level professional certification and regulatory body comparable to the ATCB in the United States or the HCPC in the United Kingdom. This means that anyone can use the title of “art therapist,” which has led to the collapse of entry thresholds, widespread confusion over qualifications, the inability of the public to distinguish professional competence, and the lack of any mechanism to regulate professional ethics. This has created the underlying cause of both the confusion surrounding practitioners' qualifications and the resulting crisis of public trust.

Secondly, the dual absence of a professional education system and public policy support has stifled the cultivation and supply of qualified professionals from the outset. In the current higher education sector, curriculum design shows a tendency toward fragmentation and de-psychologisation, with an emphasis on artistic creation and expression while neglecting core professional components such as psychological assessment, intervention techniques and clinical ethics. Teaching objectives, content and evaluation standards lack unified regulation, and the severe shortage of faculty with combined backgrounds in clinical psychology and art therapy has led to highly uneven training quality (Li, 2024). At the policy level, art therapy has not yet been officially recognized as a legitimate form of psychological support or intervention, nor has it been incorporated into public healthcare services or medical insurance schemes. This long-term lack of policy support has allowed commercial and market logics to dominate the field, further intensifying its de-professionalization (i.e., the erosion of professional standards and boundaries) and conceptual distortion. It has also generated widespread misunderstanding and misinformation in public perception, making it difficult for art therapy to be regarded as a professional discipline.

Finally, the combined effect of cultural perceptions and market forces has further entrenched conceptual confusion. At the socio-cultural level, the stigmatization of “therapy,” such as the negative labeling of people with mental illness (Li et al., 2008), and the spiritualized and aestheticized imagination of “healing” have unintentionally encouraged the substitution of terminology and the dilution of concepts. At the same time, the enormous demand for mental health support has rapidly generated an immature market. “Fast-consumption” style art healing workshops have expanded quickly because they can meet the consumer demand for emotional relief in the short term. This has, in practice, further crowded out the development pathway of professional art therapy, which requires long-term investment and evidence-based validation, and has led the entire field into a vicious cycle of low-level repetition in both conceptual definition and practical standards.

It is therefore evident that conceptual confusion and institutional deficiency have not only undermined the professional nature of art therapy but have also made it difficult to establish public trust and institutional safeguards at the community level. This has limited its accessibility and sustainable development among wider populations and has prevented it from fulfilling its intended role in providing public psychological support and fostering social integration within communities. Only through institutional development that clarifies concepts and provides informed guidance to strengthen the existing system can art therapy be enabled to realize its public value at the community level.

## 3 Toward institutionalization: clarifying concepts and building future pathways for community art therapy in China

The analysis in the preceding section suggests that the conceptual confusion pervasive in China cannot be adequately explained as a simple misunderstanding or misapplication of terminology. Rather, it arises from the interaction between globally observed theoretical fluidity and China's local institutional deficiencies. International scholarship widely recognizes that the categories of community art, arts in health and community art therapy are inherently fluid and contested (Huss, 2013; Kapitan, 2010; Shefi et al., 2022). In Western contexts, however, this fluidity is mediated and stabilized by professional infrastructures such as certification systems, ethical codes, and standardized training, which allow conceptual debates to unfold without undermining professional boundaries.

In China, by contrast, the absence of such infrastructures means that this same theoretical fluidity leads not to productive debate but to conceptual conflation, in which “healing” becomes a catch-all category obscuring crucial distinctions between clinical therapy, social practice, education and commerce. Addressing these challenges therefore requires moving beyond semantic clarification and toward the development of institutional foundations. The pathways proposed in this section aim to create the institutional conditions that can guide and support the field's conceptual fluidity in a constructive way.

To promote the transition of the art therapy field in China from conceptual confusion to institutional development, it is first necessary to clarify the professional values and internationally recognized practice elements that should guide its development. Based on in-depth interviews with 32 community art therapists from 36 countries, (Goldstein Nolan and Mumpton 2024) identified six core practice elements of international community art therapy: (1) professional competence and ethical practice, (2) attention to belonging, (3) inclusiveness and accessibility, (4) responsiveness to social issues, (5) moving beyond psychodynamic frameworks and the medical model, and (6) fostering reciprocal and egalitarian relationships as well as mind–body integration and embodied participation. These elements are highly consistent with the principles of professional ethics and cultural sensitivity advocated by international professional organizations such as the AATA, BAAT and ANZACATA, and they reflect the mainstream value orientation of the international field. In China's current institutional vacuum, these internationally recognized elements also provide an essential reference point for envisioning the foundations of a future regulatory and professional system.

In comparison, art therapy in China still shows systemic gaps in professional standards, talent cultivation, social functions and service models. Table 2 compares the six core international elements with the current situation in China and categorizes the divergences into three main types: insufficient professionalization, insufficient public orientation and insufficient systemic development. The deeper causes of these gaps lie primarily in three broken links: the absence of certification and regulation, the weakness of educational provision, and the lack of policy and funding systems. In response, this paper proposes corresponding pathways for institutional development.

**Table 2:** The six core elements of international community art therapy vs. the current situation of art therapy in China.

**International element**	**Key practices in international context**	**Current situation in China**	**Type of gap**
Skilled & ethical practice	Possesses dual knowledge in psychology & art; ethical codes & supervision; systematic training	Low entry threshold, lack of national certification & unified ethical regulation	Insufficient professionalization
Attends to belonging, inclusivity & accessibility	Emphasizes accessibility & social integration for marginalized individuals & groups	Predominantly commercial & fee-based activities, weak provision of public services	Insufficient public orientation
Addresses social issues & inequities	Focuses on both individual psychological support & broader social inequities	Mainly targets individual emotional relaxation, lacks orientation toward community-level issues	Insufficient public orientation
Beyond psychodynamic frameworks & the medical model	Integrates educational, cultural, community & health-related goals	Weak interdisciplinary collaboration, fragmented projects	Insufficient systemic development
Encourages interdependent & egalitarian relationships	Emphasizes co-creation, dialogue, trust & sustained support	One-off consumer experiences, unstable relational & support networks	Insufficient systemic development
Somatic & embodied practice	Focuses on bodily awareness, emotional co-regulation & trauma-informed approaches	Emphasizes sensory or aesthetic experience, lacks therapeutic goals & competencies	Insufficient professionalization

### 3.1 Establishing a professional certification and ethical regulatory system: laying the foundation for professional trust

The current situation of chaotic entry standards and the absence of regulatory oversight is the fundamental cause of both weakened professional trust and conceptual confusion. To change this situation, it is first necessary to establish an industry-led and interdisciplinary “Chinese Art Therapists Certification Committee” to build an initial professional consensus and lay the groundwork for future inclusion in the national occupational classification system and alignment with international professional standards. Secondly, a Unified Standards for the Registration of Art Therapists should be developed, drawing on international professional standards such as those of the ATCB. These standards should set out mandatory requirements, including educational background, credits in core psychology courses, the length of supervised practice, and ongoing supervision obligations. In addition, a code of ethics for art therapists should be established, with reference to professional codes such as the AATA. This code should institutionalize core ethical principles such as informed consent, confidentiality and privacy, avoidance of dual relationships, trauma-informed and culturally sensitive practice, risk management, and honesty and fairness, alongside an independent system for ethical oversight, complaints and disciplinary procedures.

These measures would clearly define the professional boundaries of qualified practitioners and provide a basis for building public trust. This emphasis on professional competence and ethical governance aligns with social art therapy scholarship, which underscores that community-based arts practices can sustain their therapeutic integrity only within clear ethical, supervisory, and institutional frameworks (Huss and Bos, 2018; Huss and Havsteen-Franklin, 2023).

### 3.2 Building an interdisciplinary education system: cultivating professionals with psychological competence

Institutional development relies on a high-quality talent supply, and building an interdisciplinary education system is therefore a crucial starting point for advancing the development of community art therapy in China. First, standardized training programs should be established in universities. The curriculum should cover modules such as psychological assessment and basic intervention, trauma-informed practice and crisis intervention, ethics and risk management, cultural and linguistic competence, community health and program evaluation, as well as research methods and evidence-based practice. These components are essential to ensure that students acquire solid foundations in psychology and professional ethics. Second, a systematic platform for supervised practice should be developed. Universities can collaborate with mental health centers, hospital psychology departments, community service centers, schools and eldercare institutions to establish practicum sites. Requirements should be specified for the duration of supervised practice and the qualifications of supervisors, enabling students to gain real service experience under the guidance of senior psychologists or certified art therapists. In addition, it is necessary to strengthen local cultural competence during internships and case seminars. Students should be guided to understand community mental health issues and service needs in the Chinese socio-cultural context, and to cultivate a professional orientation of “helping people before art.” Through the construction of such a system, the quality of talent cultivation can be improved from the outset. By first establishing psychological and ethical foundations and then developing skills in the use of artistic media, this approach can effectively reduce the widespread conceptual confusion currently found in the field.

### 3.3 Integrating into public policy and community health networks: promoting sustainable development

Without inclusion in public systems, art therapy will remain confined to the consumer market and will be unable to realize its intended public value at the community level. Embedding art therapy into policy frameworks is therefore the ultimate safeguard for institutionalization, and this process can be advanced in gradual stages. In the short term, pilot projects can be launched at the local level. Through government procurement of services, community art therapy can be incorporated into pilot schemes under “community health” or “mental health service packages,” with a focus on vulnerable groups such as older adults, left-behind children and children with autism. These projects can help establish initial service models and build public awareness. In the medium term, once professional certification and the talent supply have reached a basic level of maturity, the Ministry of Human Resources and Social Security should incorporate the role of “art therapist” into the National Occupational Classification Catalog. This would define its job responsibilities and employment standards, create policy interfaces for positions, competencies and payment, and enable art therapists to obtain formal status and staffing support within public institutions. In the long term, after sufficient local evidence on therapeutic effectiveness and cost-effectiveness has been accumulated, art therapy could be integrated into the medical insurance reimbursement system. This would require the development of payment standards, indications and eligibility criteria, and quality indicators, as well as the establishment of stable posts and performance evaluation mechanisms at the community level to ensure service quality and sustainability. Through these phased policy measures, a service network with public orientation and accessibility can be gradually built. Public funding and policy instruments can then be used to guide qualified practitioners into non-profit settings such as schools, communities and eldercare institutions, thereby enabling the institutionalization of art therapy in China (Berman, 2011).

## 4 Conclusion

The conceptual confusion between art healing and art therapy in China is not merely a matter of terminological misappropriation, but rather an inevitable outcome of deep-rooted institutional deficiencies in practice. These deficiencies include the vacuum of professional certification and regulation, the lagging development of professional education systems, and the lack of public policy support. Together, these factors have given rise to a de-professionalized and consumer-oriented art healing market. Its proliferation has not only blurred the boundary between art-based activities and psychology-based clinical interventions but has also hindered art therapy from fulfilling its intended public value at the community level.

**Figure 1:**
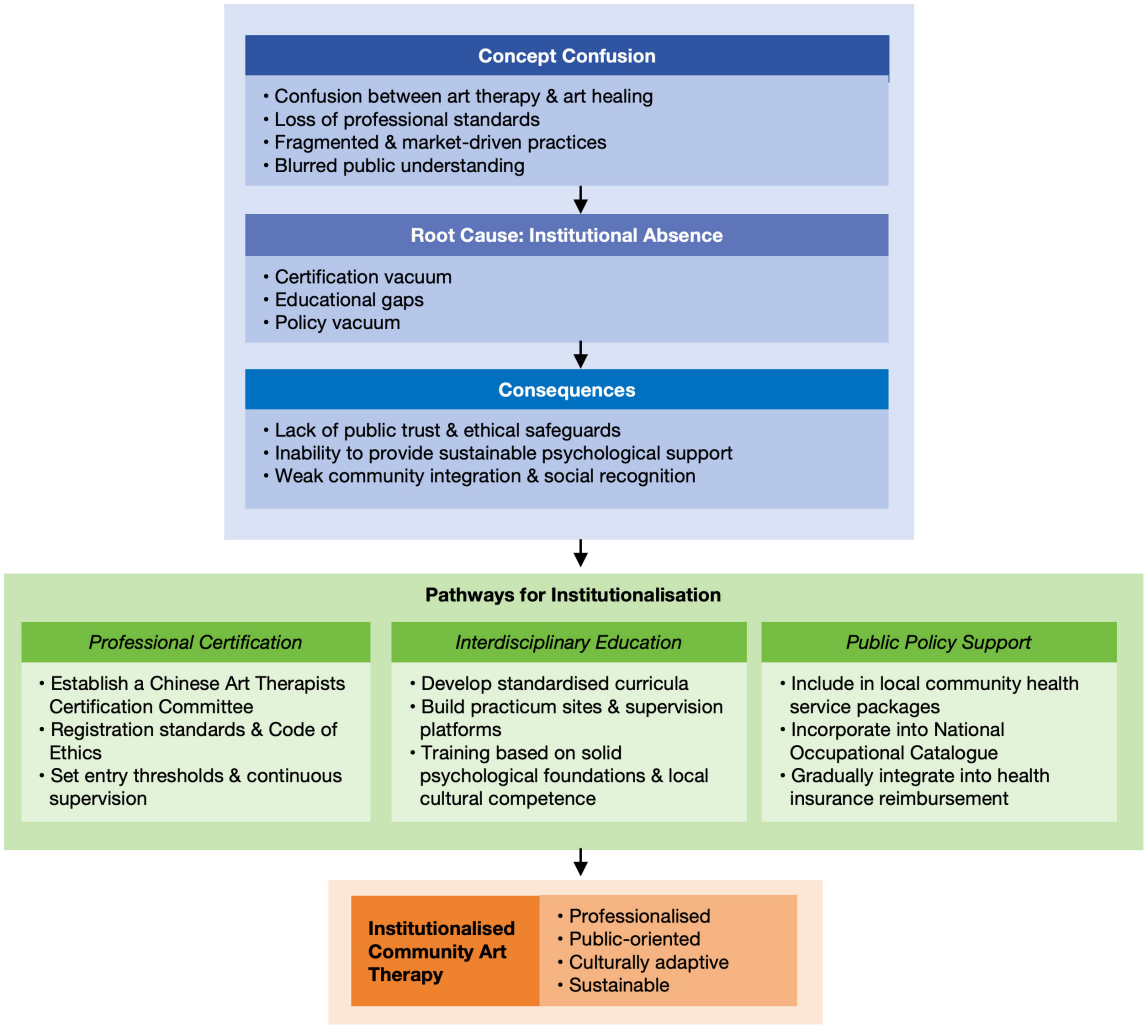
Conceptual and institutional logic of art therapy development in China: from current confusion to future institutionalization.

Achieving standardized development in this field cannot rely solely on academic debate to clarify concepts; it must be grounded in robust institutional frameworks with clear conceptual definitions. This paper argues that the development of art therapy in China requires an institutional shift. This shift involves establishing a systematic framework aligned with mature international models and built on three pillars of professional certification, educational training, and public policy support. These pathways aim to move the field away from its current commercialized, individualized, and non-professional model toward a community-based mental health service system that is professional, public-oriented and culturally adaptive (see Figure 1).

This paper seeks to outline a macro-level pathway for clarifying the core issues and future directions of the field. However, this stance may not fully capture the rich, bottom-up forms of local innovation emerging in grassroots practice. Furthermore, this paper primarily draws on mature Western systems to offer a potential model for the standardized development of art therapy in China, but future research should further explore the cultural and structural compatibility between Western models and the Chinese context in order to avoid another form of contextual dislocation. The ultimate goal of art therapy in China may not be to replicate Western pathways, but to develop theoretical and practical systems rooted in local culture while learning from international standards. Building on this foundation, future research could empirically test and assess the feasibility of the institutional pathways proposed in this paper, or conduct action research in specific population groups and community settings. Such efforts could continuously promote both the professionalization and localization of art therapy in China, ultimately enabling it to benefit wider social groups and to foster long-term and sustainable practice. In addition, beyond the Chinese context, the discussion developed in this paper may offer insights for countries or regions where community art therapy is emerging without established regulatory or educational structures.
